# Proteolytic Activity of *Zingiber officinale* Extract: Interaction With Myofibrillar Proteins of Different Origins and Functional Effects in Meat Systems

**DOI:** 10.1002/fsn3.72459

**Published:** 2026-10-06

**Authors:** Alessandro Crinò, Chiara Fabrizi, Andrea Menci, Katia Liburdi

**Affiliations:** ^1^ Department of Agricultural and Forestry Sciences (DAFNE) University of Tuscia Viterbo Italy

**Keywords:** ginger protease, meat marination, myofibrillar proteins, proteolytic kinetics, sensorial analysis

## Abstract

Plant‐derived proteases are widely investigated as sustainable tools for meat tenderization; however, their efficacy depends on enzyme characteristics, concentration, and muscle origin. This study compared a crude *Zingiber officinale* extract with bromelain and papain, two of the most established plant proteases in the food industry, integrating kinetic analysis on isolated myofibrils with technological and sensory evaluation in marinated chicken breast (CB) and pork loin (PL). Proteolytic kinetics were assessed using CB‐MF and PL‐MF at increasing enzyme protein concentrations. Based on catalytic efficiency, the most active preparations (B‐III, P‐III, ZR‐III) were selected for validation in whole muscle systems. Chicken myofibrils exhibited greater susceptibility to proteolysis than pork, while ginger extract showed substrate‐dependent behavior with preferential activity toward CB‐MF. Enzymatic marination increased myofibrillar fragmentation in both species without altering pH, confirming that structural modifications were enzyme‐driven. Ginger treatment induced rapid early‐stage fragmentation (6 h), whereas bromelain and papain promoted more extensive degradation at longer times (12 h). Cooking yield and water‐holding capacity were primarily influenced by muscle type and marination time. Chicken consistently showed higher moisture retention than pork. Ginger extract promoted controlled proteolysis, preserving cooking performance relative to bromelain and papain. Sensory evaluation under optimal conditions (6 h) demonstrated enhanced juiciness, tenderness, flavor perception, and overall acceptability in ginger‐treated samples, particularly in chicken. Overall, the integrated experimental evidence identifies ginger protease as a selective and moderate proteolytic system capable of enhancing meat quality while preventing excessive structural breakdown.

## Graphical abstract

Ginger‐derived proteases were evaluated for their proteolytic activity toward isolated chicken and pork myofibrils and their effects during meat marination. The ginger extract showed a selective and moderate proteolytic activity, resulting in controlled protein degradation and improved meat quality, including tenderness, juiciness, flavor, and overall acceptability, with a more pronounced response observed in chicken than in pork.

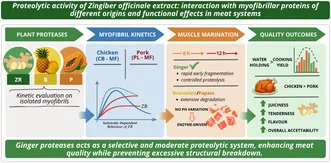

## 1 Introduction

Enzymes hold considerable potential for a wide range of innovative applications that enhance the quality, functionality, and overall value of food products. The use of enzymes in food processing was already implicitly present in ancient practices, marking the first empirical applications of biochemical principles in food technology (Tavano et al. 2018). Plant‐derived enzymes and crude plant extracts have long been employed in traditional food processes such as meat tenderization, milk coagulation, or juice clarification due to their natural abundance, safety, and remarkable technological efficiency. Their use, initially empirical, has persisted for centuries and continues to inspire modern biotechnological approaches to develop sustainable and clean‐label food innovations (Verma et al. 2022). Among the different classes of plant enzymes, proteases are among the most extensively used in the food sector due to their versatility and efficiency in modifying protein structures and improving textural properties. Well‐known examples include papain from *Carica papaya* and bromelain from pineapple, both of which are widely used as meat tenderizers (Fernández‐Lucas et al. 2017; Arshad et al. 2014). Among these plant proteases, particular attention has recently been directed toward zingibain (EC 3.4.22.67), a cysteine endopeptidase extracted from *Zingiber officinale* . This enzyme exhibits remarkable catalytic activity and selectivity toward both myofibrillar and connective tissue proteins, making it especially promising for applications aimed at enhancing the functional and textural properties of meat systems (Ahmad et al. 2020). Since zingibain primarily targets myofibrillar proteins, understanding the intrinsic composition and structural variability of muscle tissue is essential for predicting its effects across different meat systems (Ha et al. 2012; Bekhit et al. 2014). The chemical composition of meat is influenced by several biological and production factors, as well as by the intrinsic characteristics of each muscle, leading to notable variations in the distribution and structural organization of the main protein fractions (Hui 2012; Schiaffino and Reggiani 2011). Muscle proteins make up about 20% of total tissue mass and can be classified into three groups based on solubility: myofibrillar, sarcoplasmic, and stromal (Tornberg 2005). Among these groups, myofibrillar proteins, primarily myosin and actin, along with regulatory proteins such as troponins and tropomyosin, comprise the largest portion, accounting for 50%–60% of total muscle proteins (Chen et al. 2017). The composition and organization of these proteins can vary considerably among animal species and muscle types. Differences in the relative abundance and structural stability of myofibrillar components strongly affect the mechanical resistance of muscle fibers and their susceptibility to proteolysis (Bekhit et al. 2014; Chen et al. 2017). Such structural heterogeneity modulates *post‐mortem* proteolysis by endogenous proteolytic enzymes, notably the calpain system and lysosomal cathepsins, because the extent and kinetics of myofibrillar breakdown depend on both enzyme activity and the microstructural organization/accessibility of myofibrillar targets (Bhat et al. 2018; Li et al. 2023). When proteolysis during chilled aging is limited, the weakening of key cytoskeletal/myofibrillar linkages is reduced, hindering the relaxation of muscle fibers and resulting in insufficient tenderization and perceptible deterioration of sensory attributes (Bekhit et al. 2014; Hopkins and Thompson 2002). Understanding how exogenous proteases interact with myofibrillar substrates of different animal origins is therefore essential to characterize their proteolytic activity and substrate specificity, as well as to optimize their technological application in meat processing. However, this aspect has received limited attention so far and remains relatively unexplored in literature. In this context, the present study investigated the effect of *Z. officinale* Roscoe extract on chicken breast and pork loin muscles, selected as models with distinct myofibrillar structures and functional properties. The primary objective was to characterize the enzymatic activity of the extract at varying enzyme‐to‐substrate ratios and to evaluate its influence on myofibrillar proteolysis and the resulting changes in meat tenderness. A comprehensive approach integrating physicochemical analyses, structural assessments, and sensory evaluations was employed to elucidate how ginger‐derived proteases interact with muscle proteins of different animal origins and to clarify their potential as natural biotechnological tools for improving meat quality.

## 2 Material and Methods

### 2.1 Materials

#### 2.1.1 Plant and Meat Origins

For the experiment, *Z. officinale* Roscoe (Zingiberaceae) (ZR) was purchased from Unicoop Tirreno (Viterbo, Italy). The taxonomic identity and accepted botanical nomenclature of the species were verified using Plants of the World Online (POWO 2026). Chicken breast (CB) and pork loin (PL) were purchased from Unicoop Tirreno (Viterbo, Italy). Commercial chicken breast and pork loin cuts were selected based on the absence of visible defects, discoloration, and excessive exudate. Chicken breast samples were obtained from broilers slaughtered at approximately 45 days of age, whereas pork loin samples were obtained from pigs slaughtered at approximately 6 months of age. Samples were transported under refrigerated conditions and stored at 4°C until analysis. The initial pH values were 5.86 and 5.40 for chicken and pork, respectively. To support comparison between the two meat systems, storage conditions, sample preparation, marination protocol, cooking treatment, and analytical procedures were standardized. CB and PL samples were collected within 10–15 min post‐slaughter, transferred into polyethylene bags, and stored at 4°C for 24 h.

#### 2.1.2 Chemicals

All reagents used in the experimental design, including bromelain (B) from *Ananas comosus* (EC 3.4.22.32), papain (P) from *Carica papaya* latex (EC 3.4.22.2), azocasein (AzC), sodium phosphate monobasic (NaH_2_PO_4_), sodium phosphate dibasic (Na_2_HPO_4_), L‐cysteine (C_3_H_7_NO_2_S), Coomassie Brilliant Blue, trichloroacetic acid (TCA, C_2_HCl_3_O_2_), sodium hydroxide (NaOH), citric acid (C_6_H_8_O_7_), Trizma hydrochloride (C_4_H_11_NO_3_·HCl), potassium chloride (KCl), EDTA (C_10_H_16_N_2_O_8_), calcium chloride (CaCl_2_), sodium acetate (CH_3_COONa), and acetic acid (CH_3_COOH), were purchased from Merck KGaA (Milan, Italy).

### 2.2 Methods

#### 2.2.1 Preparation of Crude Extracts

The preparation of the crude enzymatic extract from *Z. officinale* Roscoe (ZR) was performed following the procedure described by Gagaoua et al. (2015), with minor modifications. Fresh ginger rhizomes were peeled and homogenized using a laboratory mixer (Philips HR 2068). Three extraction ratios, corresponding to increasing amounts of fresh ginger tissue, were applied by mixing 100, 200, or 300 g of fresh weight (g_FW_) with a constant volume of 450 mL of extraction buffer, yielding solid‐to‐liquid ratios of 0.2, 0.4, and 0.7 g·mL^−1^, respectively; these preparations were referred to as ZR‐I, ZR‐II, and ZR‐III. The extraction buffer consisted of 50 mM phosphate buffer (pH 7.0), prepared from NaH_2_PO_4_ and Na_2_HPO_4_ in appropriate proportions, and supplemented with 10 mM L‐cysteine. Each mixture was continuously stirred for 45 min at 4°C, then sequentially filtered through cheesecloth followed by Whatman No. 42 filter paper (ϕ125 mm). The resulting filtrate was centrifuged at 4500 rpm for 15 min at 4°C using a Megafuge 16R TX‐200 centrifuge (Thermo Scientific).

The total protein content of the plant‐derived extracts was subsequently determined by the Bradford assay, using bovine serum albumin (BSA) as the calibration standard (Bradford 1976).

#### 2.2.2 Extraction of Myofibrillar Proteins

Myofibrillar proteins (MF) were extracted from chicken breast and pork loin according to the procedures described by Qihe et al. (2006) and Zhao et al. (2012), with minor adaptations. Chicken breast (CB) and pork loin (PL) muscles were finely minced, and 2 g of tissue were homogenized in 40 mL of 50 mM Tris–HCl buffer (pH 7.6) containing 100 mM KCl and 5 mM EDTA. Myofibrillar proteins were collected by centrifugation at 4800 rpm for 15 min at 4°C. After discarding the supernatant, the resulting pellet was resuspended in 40 mL of the same buffer. This resuspension and centrifugation step was repeated five times to remove sarcoplasmic proteins. After the fifth wash, the myofibrillar suspension was filtered through cheesecloth to eliminate residual connective tissue and subsequently centrifuged again under the same conditions. The pellet was then washed with 40 mL of 1 M KCl solution and subjected to two additional centrifugation cycles at 4800 rpm for 15 min at 4°C. Following the final centrifugation, the supernatant was discarded and the purified myofibrils extracted from chicken breast (CB‐MF) and pork loin (PL‐MF) were resuspended in a final volume of 20 mL of 0.1 M KCl solution for subsequent analyses.

#### 2.2.3 Kinetic Proteolysis of Myofibrillar Proteins

The proteolytic activity of crude ginger rhizome extracts (ZR‐I, ZR‐II, and ZR‐III) was evaluated using myofibrils extracted from chicken breast (CB‐MF) and pork loin (PL‐MF) as substrates. Proteolysis of the two myofibrillar substrates was carried out according to the protocol described by Liburdi et al. (2026). To provide a comparative framework for interpreting the enzymatic behavior of ginger extracts, additional assays were performed using two commercial plant‐derived proteases, bromelain (B) and papain (P), which were included as reference controls. To allow a rigorous comparison among the different proteolytic systems, the protein concentration (PC) of the commercial protease preparations was adjusted to match that of the corresponding ginger crude extracts. Specifically, all enzymatic systems were standardized to three defined PC levels, expressed as 0.4, 0.7, and 1.1 mg_BSA_·mL^−1^. At each concentration level, bromelain, papain, and ginger extracts were tested in parallel and identified as B‐I, P‐I, and ZR‐I (0.4 mg_BSA_·mL^−1^), B‐II, P‐II, and ZR‐II (0.7 mg_BSA_·mL^−1^), and B‐III, P‐III, and ZR‐III (1.1 mg_BSA_·mL^−1^), respectively.

For the kinetic assays, 200 μL of each plant protease solution (B‐I/B‐II/B‐III, P‐I/P‐II/P‐III and ZR‐I/ZR‐II/ZR‐III) were diluted in 1 mL of 0.5 M Tris–HCl buffer (pH 6.0) containing 1 mM CaCl_2_. The enzymatic solutions were incubated with 10, 20, and 40 mg of myofibrillar proteins at 4°C under continuous agitation for 1 h. For the negative control (blank), 200 μL of distilled water was added in place of the enzymatic solution. The proteolytic reaction was terminated by the addition of 2 mL of TCA solution (0.11 M TCA, 0.22 M CH_3_COONa, and 0.33 M CH_3_COOH). After 20 min at 4°C, the precipitated proteins were removed by centrifugation at 4800 rpm for 15 min at 4°C. The absorbance of the resulting supernatant was measured spectrophotometrically at 275 nm using a Shimadzu UV‐2450 spectrophotometer (Shimadzu Europa GmbH).

The specific activity (SA) related to myofibrillar hydrolysis was calculated using the following formula (1):

(1) $$\mathrm{SA}=\frac{\left(\Delta \mathrm{Abs}\times V_{r}\right)}{V_{e}\times {\mathrm{mg}}_{\mathrm{BSA}}\times 0.001}$$

where ΔAbs represents the difference between the sample absorbance and the blank, *V*_*f*_ is the final volume of the enzymatic reaction, and *V*_*e*_ is the volume of the enzymatic extract. SA, expressed in International Units (IU), is defined as an increase of 0.001 absorbance units at 275 nm per minute, normalized to the protein content of the extract (mg_BSA_).

#### 2.2.4 Enzymatic Marination of Chicken and Pork Meat

Marination trials were conducted on chicken breast (CB) and pork loin (PL) samples to evaluate the effects of plant‐derived proteolytic extracts on the two different meat systems. Based on the catalytic efficiency observed in kinetic assays using CB‐MF and PL‐MF as substrates, only the enzyme preparations at the highest protein concentration (B‐III, P‐III, and ZR‐III) were selected for application to whole meat samples. The marination procedure was performed with slight modifications to the protocol described by Naveena et al. (2004). To minimize variability associated with enzyme penetration, chicken breast and pork loin samples were prepared with identical dimensions (3 × 3 × 3 cm) and subjected to the same meat‐to‐marinade ratio, enzyme concentrations, incubation temperature (4°C), marination times (6 and 12 h), and handling procedures. This standardized experimental design ensured that any differences observed between the two meat systems could be attributed primarily to their intrinsic structural and biochemical characteristics rather than to variations in the marination protocol. For enzymatic treatments, the marination solution consisted of 15 mL of the selected proteolytic extracts (B‐III, P‐III, or ZR‐III). Samples marinated in 15 mL of distilled water (H_2_O) were used as reference controls.

#### 2.2.5 Myofibrillar Fragmentation Index

The myofibrillar fragmentation index (MFI) was determined according to the method described by Zhao et al. (2012). For each marinated meat sample, 2 g of chicken breast (CB) or pork loin (PL) tissue was used for myofibril extraction. The resulting myofibrillar pellet was resuspended in 4 mL of 0.1 M KCl, and the turbidity of the suspension was measured spectrophotometrically at 540 nm. The MFI was then calculated according to Equation (2).

(2) $$\mathrm{MFI}=A_{540}\times 200$$

where *A*_540_ is the absorbance of the myofibrillar suspension measured at 540 nm; a factor of 200 is conventionally applied to express the MFI on a more interpretable scale.

#### 2.2.6 Determination of Cooking Yield and pH

For pH determination, 10 g of chicken breast (CB) or pork loin (PL) samples were minced and homogenized with 50 mL of cold distilled water, and the pH was measured using a digital pH meter (Hanna Instruments, HI2002, Woonsocket, USA).

The marination process was carried out for 6 and 12 h, after which the meat samples were rinsed with distilled water, gently blotted dry, and oven‐cooked (G3 Ferrari, G10094, Modena, Italy) at 75°C for 20 min. The cooked samples were subsequently analyzed for pH and cooking yield (CY, %).

#### 2.2.7 Water Holding Capacity

Water‐holding capacity (WHC) was determined according to the method described by Naveena et al. (2004) and applied to both chicken breast (CB) and pork loin (PL) samples. 20 g of minced meat were placed in a Falcon tube containing 30 mL of 0.6 M NaCl solution and stirred for 1 min using an IKA‐LOOPSTER digital stirrer. The samples were then incubated at 4°C for 15 min, stirred again, and centrifuged at 4000 rpm for 25 min. The supernatant was collected and measured, and WHC (%) was calculated as the percentage of water retained by the muscle fibers (3).

(3) $$\mathrm{WHC}=\frac{V_{r}}{V_{c}}\times 100$$

where *V*_*r*_ is the volume of liquid retained in the meat after centrifugation; *V*_*t*_ is the total volume of liquid added or present initially.

#### 2.2.8 Colorimetric Analysis

Meat color was evaluated on marinated samples using a CR‐5 colorimeter (Konica Minolta, Osaka, Japan) operating in the CIELAB color space (L*, a*, b*; illuminant D65, 10° standard observer). The instrument was calibrated prior to analysis using the white calibration plate and light trap provided by the manufacturer. Color measurements were performed through a 10 mm aperture after a blooming period of 30 min at 4°C, to allow stabilization of the meat surface color. Color analysis was conducted on chicken breast and pork loin samples marinated for 6 h, both before (raw) and after cooking (CB‐R, CB‐C, PL‐R, and PL‐C). The 6 h marination time was selected based on preliminary technological assessments, as it represented the condition providing the most balanced modification of meat properties and was therefore considered the most suitable for instrumental color analysis. For each sample, three analytical replicates were performed, with five measurements per replicate, and results were expressed as lightness (L*), redness (a*), and yellowness (b*) values. In addition, color shade descriptors associated with the CIELAB coordinates were obtained by processing the colorimetric data using the Nix Color Sensor conversion software (Nix Sensor Ltd., Hamilton, Canada), which allows standardized interpretation of color parameters.

To quantify color differences between treated samples and the corresponding control, the total color difference (Δ*E*) was calculated according to the standard CIELAB Equation (4):

(4) $$\Delta E=\sqrt{{\left(\Delta L\right)}^{2}+{\left(∆a\right)}^{2}+{\left(∆b\right)}^{2}}$$

#### 2.2.9 Sensory Analysis

The evaluation was conducted to characterize the profile of cooked chicken breast (CB) and pork loin (PL) samples subjected to marination. For each meat type, samples marinated in distilled water were used as reference controls and compared with the corresponding samples marinated with crude ginger extract (CB/H_2_O vs. CB/ZR‐III and PL/H_2_O vs. PL/ZR‐III). The panel consisted of 25 untrained assessors (16 females and 9 males), aged 25–32 years, all graduates of the Faculty of Agricultural and Environmental Sciences at the University of Tuscia (approximately 80% holding a Master's degree and 20% a Bachelor's degree), with previous experience in meat evaluation. All participants provided informed consent prior to participation, in accordance with ethical standards for research involving human subjects. Before testing, panelists attended a preparatory session aimed at defining and clarifying the descriptors to be assessed, including appearance, flavor, juiciness, tenderness, and overall acceptability, to ensure a shared understanding of each attribute. The evaluation was conducted in a single session, during which each panelist evaluated two cooked samples per meat type: one marinated in water (control) and one marinated with ginger extract. Samples were cooked at an internal temperature of 70°C ± 1°C, and all assessors evaluated both treatments under identical conditions. CB and PL samples were presented under blind conditions, without disclosure of the marination treatment applied, and were standardized in terms of shape, portion size, and serving temperature. Neutral containers were used to avoid interference with color and aroma perception. The assessment followed the principles of Quantitative Descriptive Analysis (QDA), allowing quantitative evaluation of predefined attributes.

#### 2.2.10 Statistical Analysis

All experiments were conducted using three independent biological replicates (*n* = 3), and results are reported as mean ± standard deviation. Data were analyzed by one‐way analysis of variance (ANOVA), followed by Tukey's multiple‐comparison test at *p* ≤ 0.05. Separate ANOVA models were used to evaluate differences among marination treatments within each meat type and differences between meat types subjected to the same enzymatic treatment. Statistical analyses were performed using DSTAT software (Johnson 1989).

## 3 Results and Discussion

### 3.1 Proteolytic Activity and Myofibrillar Fragmentation in Chicken and Pork Muscle

Plant‐derived proteases have therefore been investigated as meat‐tenderizing agents under mild processing conditions (Xu and Xu 2021). The proteolytic activity of aqueous plant extracts is mainly attributed to endogenous proteases, including bromelain from pineapple stems, papain from papaya latex, and zingibain from ginger rhizomes (Gagaoua et al. 2015). In particular, the activity of ginger extract is primarily associated with zingibain, a cysteine protease capable of hydrolyzing myofibrillar and connective tissue proteins (Mohd Azmi et al. 2023).

The proteolytic kinetics of bromelain (B), papain (P), and crude ginger extract (ZR) acting on myofibrillar proteins extracted from chicken breast (CB‐MF) and pork loin (PL‐MF) are reported in Figure 1. The catalytic curves illustrate the time‐dependent evolution of proteolytic activity at increasing enzyme concentration levels (B‐I/B‐II/B‐III; P‐I/P‐II/P‐III; ZR‐I/ZR‐II/ZR‐III) and allow comparison of proteolytic behavior toward myofibrils from chicken breast (CB‐MF) and pork loin (PL‐MF) origins. The overall trend of the curves reflects the typical relationship between hydrolysis rate and substrate concentration, consistent with Michaelis–Menten kinetics. At the lowest enzyme concentration (Figure 1A), the three proteolytic preparations (B‐I, P‐I, and ZR‐I) exhibit comparable kinetic trends when chicken myofibrils are used as substrates, whereas, in the presence of pork myofibrils, bromelain shows the highest proteolytic activity. At the intermediate enzyme concentration (Figure 1B), ginger extract (ZR‐II) displays a distinct behavior toward the two meat types, showing a higher affinity for chicken myofibrils, while B‐II and P‐II exhibit similar kinetic profiles on both substrates. A comparable pattern is observed at the highest enzyme concentrations (Figure 1C), where ginger extract again shows the highest proteolytic activity toward the CB‐MF substrate. Within each proteolytic system, increasing enzyme protein concentration resulted in higher proteolytic activity, although with enzyme‐specific response patterns. For bromelain and papain, the transition from low (Figure 1A) to intermediate and high concentrations (Figure 1B,C) led to a marked increase in activity, followed by a tendency toward saturation at the highest concentration, particularly evident on CB‐MF. This behavior suggests that, under conditions of elevated enzyme availability, substrate accessibility or diffusional limitations may become rate‐limiting (Feng et al. 2018). A comparable concentration‐dependent trend is also observed for the ginger crude extracts; however, the magnitude of the response differs among substrates. Specifically, the increase in proteolytic activity with rising protein concentration is substantially more pronounced on CB‐MF substrate than on pork myofibrils. This pattern indicates a preferential interaction of ginger proteases with CB myofibrillar proteins, resulting in a progressive enhancement of catalytic efficiency without abrupt changes in kinetic slope. The trends observed in Figure 1 are supported by the kinetic parameters reported in Table 1, which clarify whether changes in proteolytic behavior derive mainly from variations in maximum reaction capacity (*V*_max_), apparent substrate affinity (*K*_M_), or both. Overall, CB‐MF enabled higher *V*_max_ values, particularly for the ginger extract. In contrast, PL‐MF showed lower *K*_M_ values at higher enzyme concentrations, especially for bromelain, resulting in marked increases in catalytic efficiency (*V*_max_/*K*_M_). From a biological perspective, these differences can be partially explained by intrinsic structural and post‐mortem proteolytic dynamics of the two muscle types. In chicken muscle, disruption of myofibrillar structures, particularly Z‐disks and actin‐myosin cross‐bridges, occurs relatively rapidly, whereas in porcine muscle, similar structural weakening develops more slowly (Blanchard and Mantle 1996).

**FIGURE 1:**
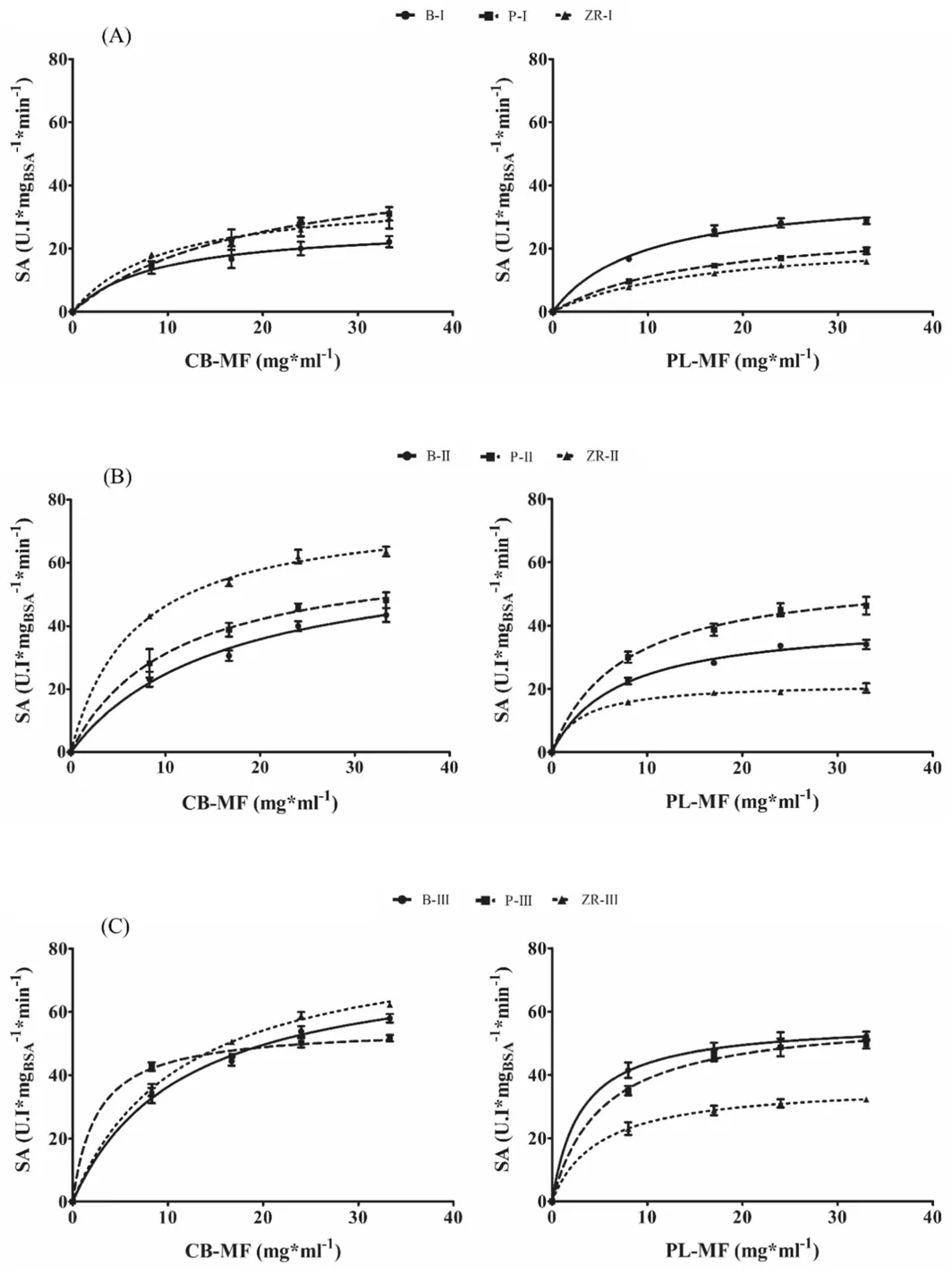
Proteolytic kinetics expressed as specific activity (SA, I.U.*mg_BSA_^−1^* min^−1^), measured using myofibrils extracted from chicken breast (CB‐MF, mg*mL^−1^) and pork loin (PL‐MF, mg*mL^−1^) as substrates. Panel A reports kinetic curves obtained with enzyme extracts standardized to 0.4 mg_BSA_*mL^−1^ protein content: Bromelain (B‐I), papain (P‐I), and crude ginger extract (ZR‐I). Panel B shows kinetics at 0.7 mg_BSA_*mL^−1^ for bromelain (B‐II), papain (P‐II), and ginger extract (ZR‐II), while panel C refers to 1.1 mg_BSA_*mL^−1^ enzyme protein concentration for bromelain (B‐III), papain (P‐III), and ginger extract (Z‐III). All reactions were carried out at 4°C in a model solution at pH 6.

**TABLE 1:** Kinetic parameters (*V*_max_, *K*_M_) and catalytic efficiency (*V*_max_/*K*_M_) of bromelain (B), papain (P), and crude ginger extract (ZR), evaluated at three enzyme protein concentrations (0.4, 0.7, and 1.1 mg_BSA_·mL^−1^, corresponding to B‐I/P‐I/ZR‐I, B‐II/P‐II/ZR‐II, and B‐III/P‐III/ZR‐III, respectively). Assays were conducted at 4°C in a model solution at pH 6 using myofibrils extracted from chicken breast (CB‐MF) and pork loin (PL‐MF) as substrates. Lowercase letters (a–c) indicate significant differences among enzymatic extracts at the same protein concentration and within the same substrate, whereas uppercase letters (A–C) denote significant differences for the same extract across increasing protein concentrations (Tukey's test, *p* ≤ 0.05). Values are expressed as mean ± standard deviation (*n* = 3).

PEs	PC (mg_BSA_/mL)	Kinetic parameters					
		CB‐MF			PL‐MF		
		*V*_max_ (U.I./mg_BSA_)	*K*_M_ (mg/mL)	*V*_max_/*K*_M_	*V*_max_ (U.I./mg_BSA_)	*K*_M_ (mg/mL)	*V*_max_/*K*_M_
B‐I	0.40	27.36^cC^ (±2.04)	8.85^bB^ (±2.09)	3.09	38.37^aB^ (±2.60)	9.51^bA^ (±1.97)	4.03
P‐I	0.40	49.14^aC^ (±2.40)	18.82^aA^ (±2.00)	2.61	28.50^bB^ (±0.44)	16.00^aA^ (±0.58)	1.78
ZR‐I	0.40	37.19^bC^ (±3.05)	9.79^bAB^ (±2.41)	3.80	24.29^cB^ (±1.11)	16.74^aB^ (±1.75)	1.45
B‐II	0.70	64.55^bB^ (± 6.53)	16.07^aA^ (± 3.79)	4.02	41.76^bB^ (±2.29)	7.07^aA^ (±1.40)	5.91
P‐II	0.70	64.65^bA^ (±2.50)	10.71^abB^ (± 1.19)	6.03	57.18^aA^ (±2.18)	7.39^aB^ (±0.99)	7.74
ZR‐II	0.70	76.96^aB^ (±2.60)	6.62^bB^ (±0.84)	11.63	21.96^cC^ (± 0.45)	3.11^bC^ (± 0.39)	7.06
B‐III	1.10	77.90^aA^ (± 3.76)	11.46^aAB^ (± 1.53)	6.80	56.91^aA^ (± 0.77)	3.04^bA^ (± 0.26)	18.72
P‐III	1.10	54.99^bB^ (± 1.48)	2.54^bC^ (± 0.50)	21.65	58.73^aA^ (± 1.70)	5.12^aB^ (± 0.66)	11.47
ZR‐III	1.10	84.77^aA^ (± 2.75)	11.36^aA^ (± 1.02)	7.46	37.11^bA^ (± 0.21)	4.90^aB^ (± 0.13)	7.57

Following the evaluation of proteolytic performance under model conditions using isolated myofibrils, kinetic behavior and related parameters were used to identify the most effective enzymatic preparations. On this basis, the extracts showing the highest catalytic efficiency toward both chicken and pork myofibrils were selected for application to the whole meat system, namely bromelain (B‐III), papain (P‐III), and crude ginger extract (ZR‐III), corresponding to the highest protein concentration tested. The transition to the real meat matrix allowed verification of enzyme‐induced structural modifications under technologically relevant conditions, while pH was continuously monitored to exclude confounding effects. Throughout marination, pH remained stable in both chicken breast (CB) and pork loin (PL), with no significant variations (*p* ≤ 0.05) observed after 6 or 12 h in any of the proteolytic solutions tested, confirming that the observed effects were attributable to enzymatic activity rather than to changes in meat acidity (Figure 2A,B). The Myofibrillar Fragmentation Index (MFI) measured on CB and PL meat after marination in water (reference control) and in the presence of the three plant‐derived proteolytic extracts were reported in Figure 3. The addition of proteolytic extracts increased MFI values in both CB and PL, with the extent of fragmentation depending on enzyme type and marination time. Overall, CB samples exhibited higher MFI values than PL under comparable conditions, indicating a greater extent of myofibrillar disruption in chicken meat. Differences among treatments were already detectable after 6 h of marination and became more evident after 12 h, particularly in samples treated with bromelain and papain. Bromelain‐ and papain‐treated samples showed a progressive increase in MFI with marination time in both meat types, reflecting extensive fragmentation of myofibrillar structures. In contrast, ginger‐treated samples displayed a distinct temporal pattern. While the overall MFI increase was lower than that observed for bromelain and papain at longer marination times, ginger extract induced the most pronounced relative increase in MFI during the first 6 h of marination in both CB and PL. The time‐dependent evolution of MFI also differed between muscle types. Fragmentation progressed more rapidly in chicken breast than in pork loin. This difference may be related not only to the susceptibility of myofibrillar proteins to enzymatic hydrolysis but also to the distribution of the enzymatic solution within the meat. Indeed, marinade diffusion can vary among muscle, connective, and adipose tissues, resulting in a non‐uniform distribution of the marinating agents (Shi et al. 2023). The results obtained with whole meat were consistent with the assays on isolated myofibrils (Figure 1), which showed greater susceptibility of chicken myofibrillar proteins and a more gradual proteolytic action of the ginger extract. Similar muscle‐dependent responses to plant proteases have been reported in previous studies, which attributed faster fragmentation in poultry meat to lower connective tissue content and reduced structural constraints compared to pork muscle (Koohmaraie and Geesink 2006; Marino et al. 2014). Moreover, the early‐stage effectiveness of ginger protease observed here is consistent with earlier findings that describe ginger as a milder tenderizing agent that primarily promotes initial myofibrillar weakening without excessive degradation during prolonged treatment (Naveena et al. 2004; Kim et al. 2007).

**FIGURE 2:**
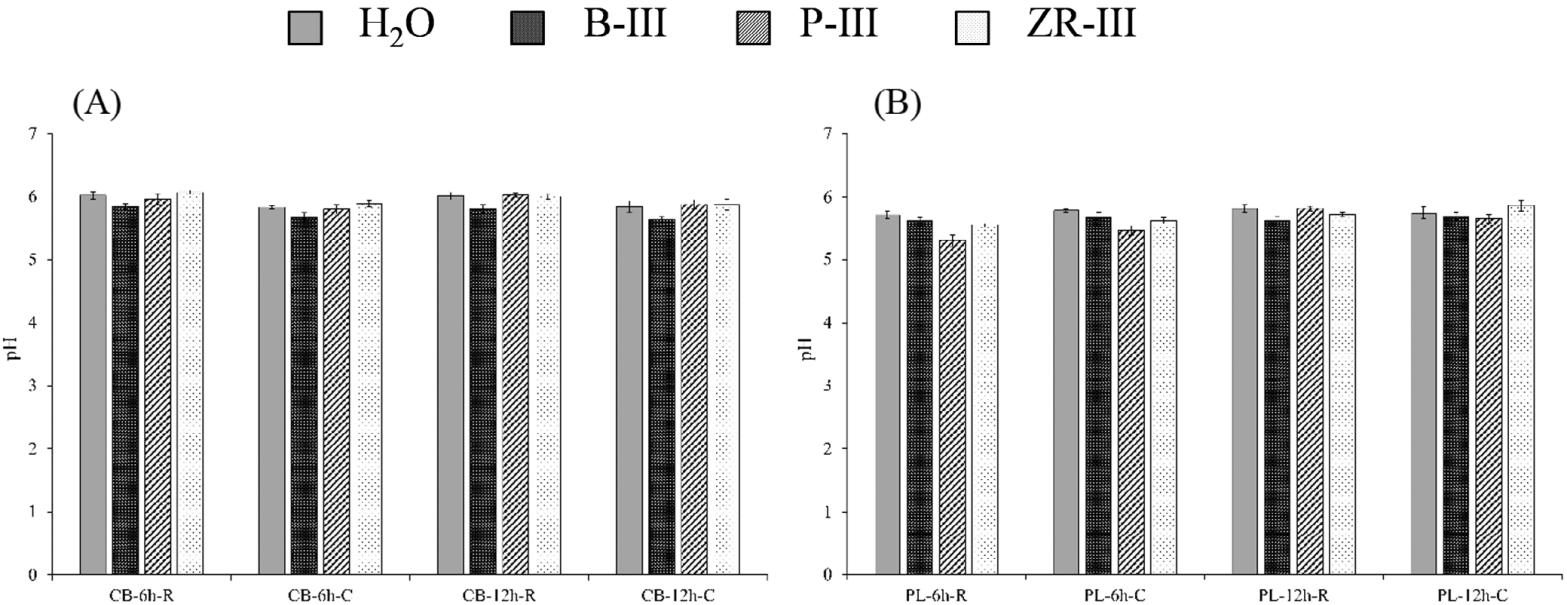
pH values measured before and after cooking in chicken breast (CB) and pork loin (PL) meat marinated in water (H_2_O), bromelain (B‐III), papain (P‐III), and crude ginger extract (ZR‐III). Panel A reports pH values for CB samples, while Panel B reports pH values for PL samples. Measurements were performed on samples marinated for 6 h (CB‐6h and PL‐6h) and 12 h (CB‐12h and PL‐12h), both in the raw (R) and cooked (C) state. Different lowercase letters indicate significant differences between raw and cooked samples of the same meat type and marination time (Tukey's test, *p* ≤ 0.05).

**FIGURE 3:**
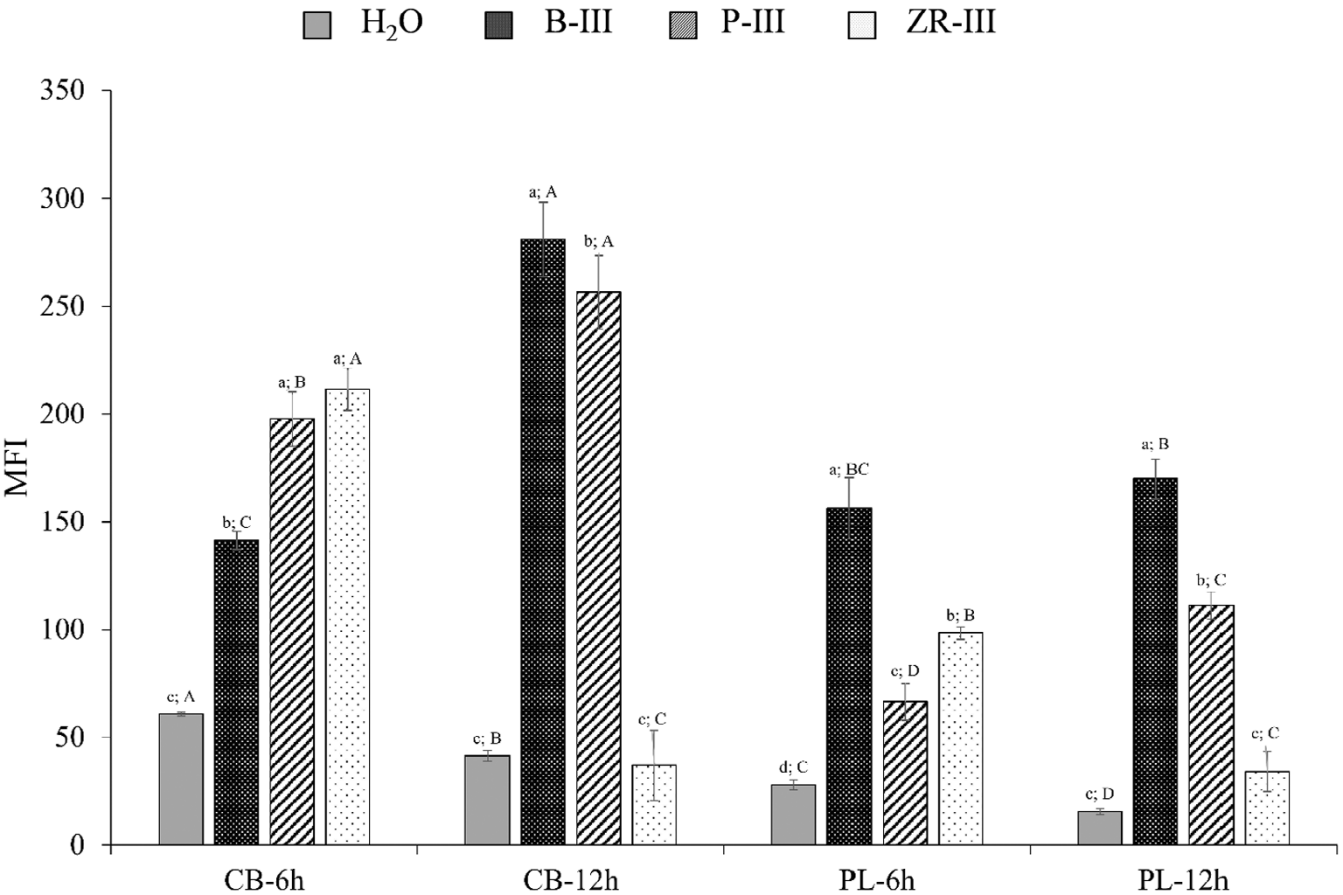
Myofibrillar Fragmentation Index (MFI) of chicken breast (CB) and pork loin (PL) meat measured on samples marinated in water (H_2_O, reference control) or in the presence of plant protease extracts bromelain (B‐III), papain (P‐III), and crude ginger extract (ZR‐III). Marination was carried out under refrigerated conditions for 6 and 12 h. Different lowercase letters (a–c) indicate significant differences among marination treatments at the same time point, whereas different uppercase letters (A–C) indicate significant differences within the same treatment between marination times (Tukey's test, *p* ≤ 0.05).

### 3.2 Effects of Marination on Water‐Holding Capacity and Cooking Yield

Marination‐induced proteolysis can substantially modify the water‐holding capacity of meat and its behavior during thermal processing. In this context, the effects of marination were assessed by analyzing water‐holding capacity (WHC) in both raw and cooked samples, together with cooking yield (CY), to elucidate how different proteolytic treatments modulated moisture retention and mass losses during cooking.

Cooking yield (CY, %) was used to assess the impact of different proteolytic treatments on water retention during thermal processing of marinated meat (Figure 4). In chicken breast samples, clear differences among marination treatments were observed at both 6 h and 12 h. At 6 h (CB‐6 h), samples treated with the ginger extract (ZR‐III) showed higher CY values than papain‐treated samples and values comparable to the water control, whereas bromelain treatment resulted in intermediate yields. After 12 h of marination (CB‐12 h), the same trend was observed, with ZR‐III‐treated samples showing the highest cooking yield (CY), higher than papain‐treated samples and comparable to or higher than the control. In contrast, papain‐treated chicken samples consistently exhibited the lowest CY values, indicating increased cooking losses and suggesting that excessive proteolysis compromised protein matrix stability during heating rather than enhancing water retention. In pork loin, CY values were generally lower than in chicken. At 6 h of marination, no significant differences were detected (*p* ≤ 0.05) among the enzymatic treatments; however, both bromelain and papain were associated with lower CY values compared with the water control. After 12 h, PL samples marinated with ZR‐III exhibited the highest CY values, which were comparable to the control and significantly higher (*p* ≤ 0.05) than those observed for the other proteolytic treatments. Overall, pork loin exhibited lower CY values than chicken breast, reflecting intrinsic structural differences between the two muscles, such as a more compact myofibrillar organization and lower water‐binding capacity in porcine muscle. Within both meat types, papain, and to a lesser extent bromelain, were associated with reduced CY, consistent with excessive proteolytic disruption of the protein network that facilitated water loss during heating. Conversely, ginger‐treated samples showed a more balanced structural modification, allowing textural improvement while preserving cooking performance. This behavior agrees with previous studies on natural plant‐based marinades, which demonstrated that controlled structural modifications can improve meat tenderness while preserving technological quality traits, including water retention and cooking performance (Yerli̇kaya et al. 2026). Comparable effects have been reported in other muscle foods. In buffalo meat, ginger and *Cucumis trigonus* extracts improved tenderness and water‐holding capacity, while ginger‐treated samples received higher sensory scores than those treated with papain (Naveena et al. 2004). In rabbit biceps femoris, papain, ginger extract, and kiwifruit extract affected water‐holding capacity and cooking loss depending on extract concentration and treatment time (Doneva et al. 2018). Papain and bromelain also reduced hardness and shear force in squid ( *Loligo vulgaris* ), although their effects on water‐holding capacity and cooking loss varied with enzyme type and concentration (Gokoglu et al. 2017). These findings indicate that the technological effects of plant proteases depend on both the enzyme and the muscle matrix (Mohd Azmi et al. 2023).

**FIGURE 4:**
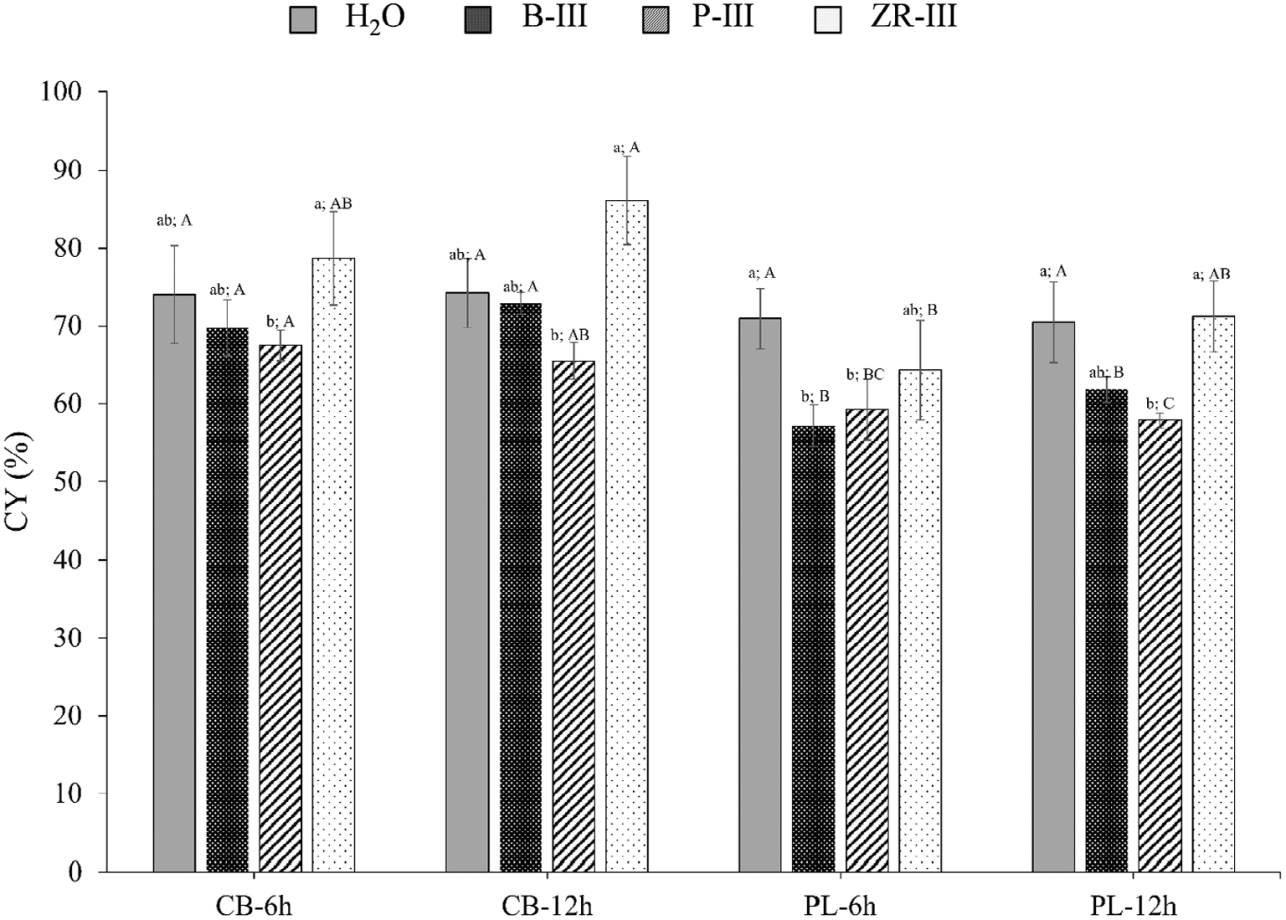
Cooking yield (CY, %) of chicken breast (CB) and pork loin (PL) meat measured on samples marinated in water (H_2_O, reference control) or in the presence of plant protease extracts bromelain (B‐III), papain (P‐III), and crude ginger extract (ZR‐III). Marination was carried out under refrigerated conditions for 6 and 12 h. Different lowercase letters (a–c) indicate significant differences among marination treatments at the same time point and within the same meat type, whereas different uppercase letters (A–C) indicate significant differences within the same treatment between marination times and between meat types (Tukey's test, *p ≤* 0.05).

Consistent with the present findings, higher CY values associated with ginger‐based treatments and lower yields in pork compared to chicken have been widely reported in the literature (Tornberg 2005; Zhao et al. 2012; Marino et al. 2014; Mendiratta et al. 2010; Abdeldaiem et al. 2014; Huff‐Lonergan and Lonergan 2005). In this context, the analysis of water‐holding capacity provides complementary information on moisture retention prior to cooking, helping to interpret differences in cooking performance. The water‐holding capacity (WHC, %) of CB and PL after marination is reported in Figure 5. This parameter was mainly influenced by marination time rather than by the proteolytic treatment applied; however, a distinct response was observed between chicken and pork meat. In chicken breast, WHC values differed among all treatments, with each proteolytic system producing a measurable and time‐dependent effect on water retention. Higher WHC values were consistently recorded after 6 h of marination, indicating improved water entrapment associated with moderate proteolysis, whereas a reduction was observed after 12 h, likely due to progressive weakening of the myofibrillar network. Such effects have been previously reported for prolonged enzymatic tenderization, where excessive proteolysis negatively affected water‐binding properties (Tornberg 2005; Kim et al. 2007). In contrast, pork loin exhibited a less differentiated response to enzymatic marination. In this meat type, samples treated with bromelain (B‐III) and papain (P‐III) did not show differences in WHC between 6 and 12 h of marination, suggesting a limited time‐dependent effect of these proteases on the water‐binding properties of porcine muscle. This behavior reflects the greater structural resistance of pork myofibrillar proteins to enzymatic modification. Overall, chicken samples consistently exhibited higher WHC values than pork at comparable marination times, reflecting the lower connective tissue content and greater myofibrillar flexibility of poultry muscle, which likely contributed to the higher CY values observed in chicken despite comparable WHC trends (Marino et al. 2014; Huff‐Lonergan and Lonergan 2005).

**FIGURE 5:**
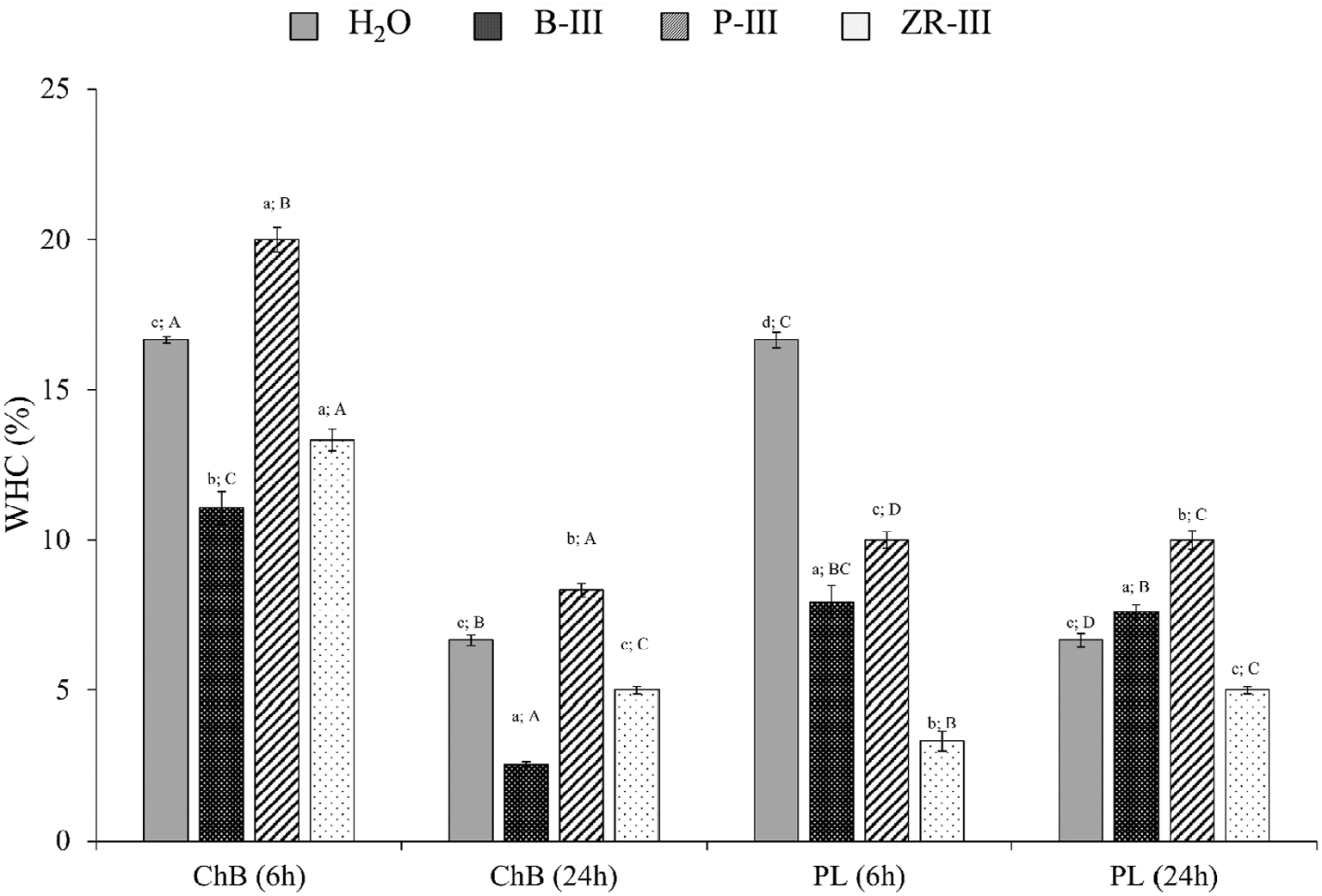
Water holding capacity (WHC, %) of chicken breast (CB) and pork loin (PL) meat measured on samples marinated in water (H_2_O, reference control) or in the presence of plant protease extracts bromelain (B‐III), papain (P‐III), and crude ginger extract (ZR‐III). Marination was carried out under refrigerated conditions for 6 and 12 h. Different lowercase letters (a–c) indicate significant differences among marination treatments at the same time point and within the same meat type, whereas different uppercase letters (A–C) indicate significant differences within the same treatment between marination times and between meat types (Tukey's test, *p ≤* 0.05).

### 3.3 Color Changes Induced by Marination and Cooking

Based on the results obtained for CY and WHC, the marination time of 6 h was identified as the most favorable condition and was therefore selected for the subsequent colorimetric evaluation of both poultry and pork samples (Table 2). CIELAB analysis was conducted on raw (R), and cooked (C) meat samples marinated in H_2_O control and in the proteolytic extracts (B‐III, P‐III, and Z‐III). In raw chicken breast samples (CB‐R), marination with the ginger extract (Z‐III) resulted in a better preservation of color intensity, particularly with respect to the a* parameter, indicating enhanced red saturation compared to water‐marinated samples and those treated with bromelain or papain. The maintenance of redness in raw poultry meat is closely linked to myoglobin stability, while reductions in a* values are commonly associated with oxidative conversion of myoglobin iron. The lower color stability observed in H_2_O, B‐III and P‐III‐treated samples may therefore be attributed to increased susceptibility to pigment oxidation, whereas the Z‐III treatment appeared to exert a protective effect, likely due to the antioxidant activity of ginger‐derived phenolic compounds (Shaukat et al. 2023). After cooking at 75°C for 20 min, chicken breast samples (CB‐C) showed pronounced treatment‐dependent changes in color parameters. Thermal processing led to an increase in lightness (L*) in control samples, consistent with protein and myoglobin denaturation. Enzymatic marination modulated this response: papain‐treated samples exhibited marked color alterations, reflected by high ΔE values, while ginger‐treated samples showed reduced L* and pronounced overall color changes, suggesting enhanced browning phenomena during heat treatment. These trends agree with previous findings reporting substantial color modifications in chicken breast meat as a function of thermal load and muscle protein denaturation (Rabeler and Feyissa 2018). In pork loin samples, raw meat (PL‐R) exhibited significant (≤ 0.05) differences in colorimetric parameters following enzymatic marination, confirming that proteolytic treatments can alter pigment environment and light scattering properties. Upon cooking (PL‐C), all samples showed an increase in L*, as expected for thermally treated pork, although the extent of color change strongly depended on the marination treatment. Papain‐treated samples displayed the highest L* and Δ*E* values, indicating extensive color modification, whereas bromelain‐treated samples remained closer to the control. Ginger‐treated samples showed comparatively higher a* values, suggesting a partial preservation of reddish tones after cooking, in line with literature data highlighting the combined effects of endpoint temperature and muscle chemistry on pork color (Lien et al. 2002). Color changes induced by ginger‐based marinades have also been observed in other meat systems. Ginger extract modified L*, a*, and b* values in camel meat, with a response influenced by the presence of citric acid (Moeini et al. 2024). Changes in color parameters following treatment with proteolytic extracts from different *Z. officinale* varieties have also been reported in beef (Liburdi et al. 2026), indicating that the color response depends on both the meat matrix and extract composition.

**TABLE 2:** CIELAB color parameters (L*, a*, b* and Δ*E*) of poultry (CB) and pork (PL) meat samples marinated for 6 h in water (H_2_O), bromelain (B‐III), papain (P‐III) and ginger extract (Z‐III), analyzed before (R) and after cooking (C). Different lowercase letters (a–c) within the same meat type and processing condition indicate significant differences among marination treatments (Tukey's test, *p* ≤ 0.05). Values are expressed as mean ± standard deviation (*n* = 3).

Meat sample	Proteolytic extract	L*	a*	b*	Δ*E*
CB‐R	H_2_O	48.91^a^ (±1.46)	‐3.07^c^ (±0.05)	3.07^c^ (±0.04)	
	B‐III	50.75^a^ (±1.32)	‐3.14c (±0.07)	4.33^a^ (±0.08)	2.49
	P‐III	46.64^a^ (±1.24)	−2.72^b^ (±0.06)	3.95^b^ (±0.07)	3.02
	ZR‐III	39.94^b^ (±1.15)	−2.03^a^ (±0.04)	2.34^d^ (±0.05)	41.04
CB‐C	H_2_O	81.1^a^ (±3.48)	‐2.05^c^ (±0.07)	13.68^a^ (±1.28)	
	B‐III	74.70^a^ (±3.71)	−4.56^d^ (±0.21)	7.22^b^ (±0.41)	44.49
	P‐III	63.31^b^ (±5.10)	−1.04^a^ (±0.10)	7.80^b^ (±0.40)	176.21
	ZR‐III	47.65^c^ (±3.83)	−1.48^b^ (±0.19)	6.73^b^ (±0.24)	585.46
PL‐R	H_2_O	46.17^bc^ (±3.46)	1.20^b^ (±0.05)	5.96^a^ (±0.34)	
	B‐III	49.50^b^ (±2.52)	0.26^d^ (±0.05)	5.99^a^ (±0.25)	5.98
	P‐III	56.35^a^ (±4.67)	0.58^c^ (±0.15)	2.35^c^ (±0.10)	58.52
	ZR‐III	44.47^c^ (±3.24)	2.07^a^ (±1.02)	3.90^b^ (±0.36)	3.94
PL‐C	H_2_O	56.93^b^ (±4.69)	0.49^b^ (±0.06)	9.52^a^ (±0.05)	
	B‐III	61.15^ab^ (±4.28)	0.10^d^ (±0.21)	9.58^a^ (±0.85)	8.95
	P‐III	69.07^a^ (±3.1)	0.23^c^ (±0.12)	3.72^c^ (±0.32)	90.45
	ZR‐III	55.27^b^ (±3.16)	0.88^a^ (±0.04)	6.46^b^ (±0.45)	6.34

Overall, enzymatic marination affected color attributes in both raw and cooked meat samples, with thermal processing enhancing treatment‐related differences. Similar heat‐dependent color changes associated with protein and myoglobin denaturation have been reported for chicken breast and pork loin (Rabeler and Feyissa 2018; Lien et al. 2002). Within this context, the color response of ginger‐marinated samples after cooking motivated their selection for subsequent sensory evaluation.

### 3.4 Sensory Attributes of Cooked Marinated Meat

Based on the identification of the most effective marination conditions through combined MFI, CY, and WHC outcomes, sensory evaluation was performed on chicken breast (CB) and pork loin (PL) samples marinated for 6 h with the crude ginger‐derived extract (ZR‐III) and subsequently cooked at 75°C for 20 min. Samples marinated in water (H_2_O) were used as reference controls for each meat type. This targeted approach enabled sensory assessment under technologically optimal conditions while minimizing variability associated with less effective treatments. The sensory results are summarized in the heatmap reported in Figure 6. Overall, ginger marination positively influenced the sensory profile of both meat types, with higher scores compared to the corresponding water‐marinated controls. The effect was more evident in chicken breast, where ZR‐III marination led to markedly higher scores for odor intensity, flavor, sapidity, and overall acceptability. Ginger‐treated chicken also exhibited increased chewiness and juiciness, reflecting a clear sensory impact of the controlled proteolytic action on texture and taste attributes. In pork loin, ZR‐III marination led to improvements in odor intensity, flavor, juiciness, tenderness, and overall acceptability relative to the control, with statistically significant (*p* ≤ 0.05) differences observed for odor intensity, flavor, and overall acceptability. However, the magnitude of these changes was generally lower than that observed in chicken, consistent with the greater structural resistance of porcine muscle to enzymatic modification. Across both meat types, ginger‐derived aroma was positively perceived and did not introduce undesirable sensory notes, while metallic/ferrous perceptions tended to be reduced in ginger‐marinated samples.

**FIGURE 6:**
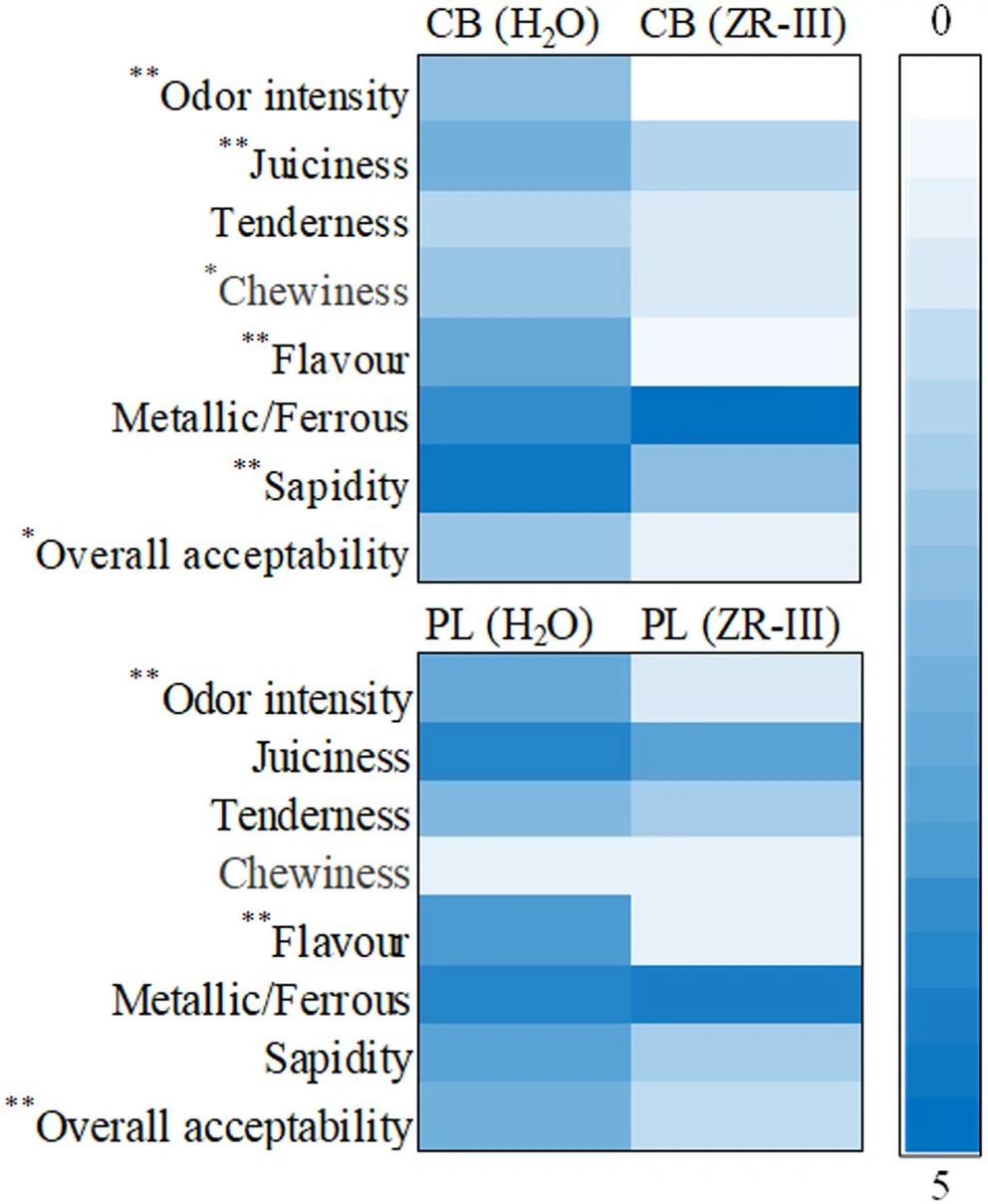
Sensory profile of cooked chicken breast (CB) and pork loin (PL) samples marinated in water (H_2_O) or crude ginger extract (ZR‐III). Data are presented as a heatmap of mean sensory scores for each descriptor (5‐point scale), where color intensity ranges from white (lowest score) to dark blue (highest score). Statistical differences between H_2_O and ZR‐III treatments within each meat type were assessed by Tukey's test: *p ≤ 0.05; **p ≤ 0.01.

Overall, the sensory responses were consistent with the instrumental results, as water retention and controlled myofibrillar modification were associated with improved juiciness and tenderness (Tornberg 2005; Huff‐Lonergan and Lonergan 2005). A recent study on Korean black goat *biceps femoris* also showed that ginger extract reduced the perception of goatiness and improved sensory scores for chewiness and overall acceptability, with the best response obtained at an extract concentration of 7% (An and Kim 2024). These findings indicate that ginger‐based marination can improve sensory quality in different meat matrices, although the response depends on muscle type and treatment conditions.

## 4 Conclusions

This study demonstrates that plant‐derived proteolytic systems differentially affect myofibrillar structure, technological properties, color stability and sensory attributes of chicken breast and pork loin, with outcomes strongly dependent on enzyme source, marination time and muscle type and origin. Proteolytic kinetics and MFI data confirmed a higher susceptibility of chicken muscle to proteolytic degradation compared to pork, reflecting intrinsic differences in muscle organization and post‐mortem proteolytic dynamics. The combined evaluation of WHC and CY indicates that cooking performance is governed not only by pre‐cooking water retention but also by the thermal stability of the protein matrix. In this context, ginger‐based marination promoted a controlled proteolytic action that preserved structural integrity during heating, resulting in improved cooking yield and stable color attributes, particularly at a marination time of 6 h. Sensory evaluation under these conditions further indicated enhanced juiciness, tenderness, and overall acceptability in both meat types treated with rhizome extracts.

Overall, the results identify crude ginger extract as a promising natural marination agent capable of modulating meat quality through balanced proteolysis, supporting its potential application in meat processing strategies aimed at improving product performance while maintaining sensory appeal.

## Author Contributions

**Alessandro Crinò:** methodology, resources, validation, writing – original draft, writing – review and editing. **Chiara Fabrizi:** methodology, writing – original draft. **Andrea Menci:** investigation, formal analysis. **Katia Liburdi:** conceptualization, validation, writing – review and editing, supervision.

## Ethics Statement

Participants in the sensory evaluation provided written informed consent prior to participation. The study did not involve any invasive procedures or collection of health‐related data; therefore, ethical approval was not required according to the institutional policy. All data were anonymized and participation was entirely voluntary.

## Conflicts of Interest

The authors declare no conflicts of interest.

## Data Availability

The data that support the findings of this study are available from the corresponding author upon reasonable request.
